# Advanced Photocatalytic Nanomaterials for Energy Conversion and Environmental Remediation

**DOI:** 10.3390/nano13152246

**Published:** 2023-08-03

**Authors:** Junying Zhang, Yong Chen, Jungang Hou

**Affiliations:** 1 School of Physics, Beihang University, Beijing 100191, China; 2 Technical Institute of Physics and Chemistry, Chinese Academy of Sciences, Beijing 100190, China; chenyong@mail.ipc.ac.cn; 3 School of Chemical Engineering, Dalian University of Technology, Dalian 116024, China; jhou@dlut.edu.cn

With the rapid development of the economy and society, the problem of energy shortage and environmental pollution is receiving more and more attention. Researchers worldwide have been exploring new technologies to produce clean energy and decrease pollution. Photocatalytic technology is one of the most promising long-term solutions for producing valuable chemical fuels and degrading pollutants directly from green solar energy. Photocatalytic materials, most of which are nanomaterials, have become a research hotspot in the field of materials.

This Special Issue compiles eleven articles dedicated to photocatalysts, including six on hydrogen production, four on organics degradation, and one combining both aspects. A good photocatalyst must meet two conditions: a broad range of light absorption and a high utilization rate of photogenerated charge-carriers. In general, most photocatalytic semiconductors can only work under violet and short-wavelength light because of their wide band gaps. In addition, the electrons and holes on a single photocatalyst exhibit a high recombination rate, leading to low utilization of the photo-generated charges. As a result, the photocatalytic performance of a single material is often very poor. These eleven papers were devoted to achieving high-efficiency photocatalysts by broadening the light absorption range, inhibiting the photo-induced electrons and holes recombination, and promoting the extraction of photo-induced charges for chemical reactions. The strategies include defect engineering [1], element doping [2,3], morphology manipulation [3,4], and binary and ternary composite fabrication [5,6,7,8,9,10,11]. Herein, we will provide a brief introduction to these works.

Defect engineering and element doping can modulate the band structure of a semiconductor and have, thus, been widely used to broaden the light absorption range and improve the utilization of photo-generated charges of photocatalytic materials. A series of black TiO_2_ was prepared by Zhou et al. [1] by altering the calcination atmospheres of the samples. Their results show that a vacuum atmosphere favors the formation of oxygen vacancies in TiO_2_, which leads to better visible-light adsorption, a narrower bandgap, and higher photo-induced charge separation, thus achieving a higher photocatalytic degradation capacity for methylene blue (MB) in comparison with inert atmospheres (He and N_2_). Using density functional theory (DFT) calculation, Jaramillo-Fierro et al. [2] predicted that La could be thermodynamically stably incorporated on the surface of ZnTiO_3_ and decrease the band gap from 3.16 eV to 2.92 eV. Furthermore, MB adsorption on the La/ZnTiO_3_ surface is much easier than on ZnTiO_3_, which indicates that introducing La will increase the photocatalytic MB degradation ability of ZnTiO_3_. Lu et al. [3] prepared an N, P self-doped carbon material with unique surface wrinkling and a hierarchical porous structure. This special morphology, together with N, P self-doping, improves the light absorption and the charge-carrier separation/transfer efficiency, and, thus, enhances the photocatalytic hydrogen production performance.

When two or three semiconductors form a composite, charge transfer usually occurs at the interface, which generally promotes the separation of electrons and holes. As a result, forming binary and ternary composites can also enhance the photocatalytic activity by increasing the utilization efficiency of the photo-generated charge-carriers, aside from broadening light absorption. Chou et al. [4]. synthesized a honeycomb-like BiFeO_3_/g-C_3_N_4_ composite, which had a much better photocatalytic ability to degrade rhodamine B (RhB) than pristine g-C_3_N_4_ and BiFeO_3_. The unique morphology facilitates the diffusion of the reactants and the utilization of light, while the polarized BiFeO_3_ surface tightly binds the dye molecules. Furthermore, the Z-scheme heterojunction and the ferroelectric effect synergistically promote the separation and migration of the photo-generated charges. Dang et al. [5]. prepared a binary type-II heterojunction photocatalyst from g-C_3_N_4_ and K_7_HNb_6_O_19_. The type-II heterojunction induces interfacial charge transfer and inhibits the recombination of photo-generated electrons and holes, hence displaying an excellent hydrogen evolution rate. Cabot et al. [6] synthesized Cu_2_O-TiO_2_ type-II heterojunctions for photocatalytic ethanol dehydrogenation. Adding Cu_2_O extends the light absorption towards the visible range, and charge transfer on the interface promotes the utilization of photo-generated charges. Hence, the Cu_2_O-TiO_2_ exhibits a hydrogen evolution rate tenfold higher in comparison with bare TiO_2_. Wang et al. [7] reported a plasma Ag-modified α-Fe_2_O_3_/g-C_3_N_4_ S-Scheme heterojunction that performed much more efficiently than the original g-C_3_N_4_ in terms of photocatalytic degradation of tetracycline and hydrogen production. The introduction of Ag nanoparticles expands the light absorption and induces high-energy electrons and photothermal effects from the local surface plasmon resonance effect, which leads to improved photocatalytic activity with joint contributions from the 2D/2D S-Scheme induced electron–hole separation. Kim et al. [8] deposited narrow-bandgap Ag_3_PO_4_ on flower-like TiO_2_@Ti_3_C_2_ to obtain a ternary composite with broad-range visible-light absorption, a high surface area, and efficient separation of photo-generated electrons and holes. The Ag_3_PO_4_-deposited TiO_2_@Ti_3_C_2_ is able to efficiently degrade organic dyes, including RhB, MB, crystal violet (CV), and methylene orange (MO), under solar light irradiation.

Photocatalytic semiconductors usually lack active sites, so co-catalysts have been extensively used to promote the photocatalytic activity. They extract photo-generated charge-carriers from the photocatalysts and offer active sites for the chemical reactions. Zhang et al. [9] selectively anchored a Pt co-catalyst to specific nitrogen species on the surface of g-C_3_N_4_ to achieve highly dispersed homogeneous Pt nanoparticles containing Pt^0^ and Pt^2+^ species. In comparison with the non-selectively deposed Pt nanoparticles, the highly dispersed Pt co-catalyst performs significantly better in promoting the photocatalytic hydrogen production of g-C_3_N_4_. Cai et al. [10] deposited a CoH_x_PO_y_ co-catalyst on CdIn_2_S_4_ nanosheets. The CoH_x_PO_y_ accelerates the hole transfer and inhibits the electron and hole recombination, thus promoting the photocatalytic hydrogen evolution reaction activity under visible light. CoH_x_PO_y_ nanoparticles exhibit a better hydrogen evolution reaction improvement effect on the CdIn_2_S_4_ photocatalyst than Pt nanoparticles. Wang et al. [11] prepared porous hollow spherical Pd/CdS/NiS, wherein the Pd and NiS co-catalysts were loaded inside and outside the hollow CdS shell layer. This special structure spatially separates the photo-generated electrons and holes, therefore remarkably increasing the photocatalytic hydrogen production rate of CdS under visible light.

In summary, the works reported in these eleven papers cover various aspects of photocatalytic materials from both theoretical calculation-based and experimental perspectives. In these works, measures of introducing dopants, creating defects, modulating morphology, and forming composite materials are proposed in order to broaden the optical absorption and promote the utilization rate of photo-generated charges, aiming to enhance photocatalytic organics degradation and hydrogen production. These strategies will have important reference value for the development of high-efficiency photocatalytic materials with wide spectral response, and, thus, will be of broad interest to general readers.
